# Application of Three-Dimensional Printing Technology in the Perioperative Management of Cardiac Tumours: A Review and Analysis

**DOI:** 10.31083/j.rcm2503101

**Published:** 2024-03-11

**Authors:** Huan Wang, Jixiang Liang, Gen Zhang, Dongsheng He, Baoluo Du, Zhipeng Ren, Ziqiang Dai, Hsin Lee, Dianyuan Li

**Affiliations:** ^1^Department of Cardiovascular Surgery, The Affiliated Suzhou Hospital of Nanjing Medical University, Suzhou Municipal Hospital, Gusu School, Nanjing Medical University, 215008 Suzhou, Jiangsu, China; ^2^The State Key Laboratory for Manufacturing Systems Engineering, Xi’an Jiaotong University, 710000 Xi’an, Shaanxi, China

**Keywords:** review, three-dimensional printing, cardiac tumours, perioperative management

## Abstract

**Background:**

Multimodal imaging plays a crucial role in evaluating suspected cardiac tumours. In recent years, three-dimensional (3D) printing technology has continued to advance such that image-based 3D-printed models have been incorporated into the auxiliary diagnosis and treatment of cardiac tumour diseases. The purpose of this review is to analyze the existing literature on the application of 3D printing in cardiac tumour surgery to examine the current status of the application of this technology.

**Methods:**

By searching PubMed, Cochrane, Scopus and Google Scholar, as well as other resource databases, a completed review of the available literature was performed. Effect sizes from published studies were investigated, and results are presented concerning the use of 3D surgical planning in the management of cardiac tumours.

**Results:**

According to the reviewed literature, our study comes to the point that 3D printing is a valuable technique for planning surgery for cardiac tumours. As shown in the review report, Mucinous and sarcomatous tumours are the most commonly used tumours for 3D printing, magnetic resonance imaging (MRI) and computed tomography (CT) are the most commonly used technologies for preparing 3D printing models, the main printing technology is stereolithography, and the most used 3D modeling software is Mimics. The printing time and cost required for 3D printing are affected by factors such as the size of the type, complexity, the printed material and the 3D printing technology used. The reported research shows that 3D printing can understand the anatomy of complex tumour cases, virtual surgical simulation, as well as facilitate doctor-patient communication and clinical teaching.

**Conclusions:**

These results show that the development of 3D printing technology has brought more accurate and safe perioperative treatment options for patients with cardiac tumours. Therefore, 3D printing technology is expected to become a routine clinical diagnosis and treatment tool for cardiac tumours.

## 1. Introduction

Cardiac tumours refer to neoplastic growths that develop in the myocardium or adjacent tissues, which can be categorised as either primary or secondary (metastatic tumour). Autopsy studies have shown that cardiac tumours have an incidence rate of 0.02%, with 75% and 25% of the cases being benign and malignant respectively. Myxomas are the most common type of benign primary cardiac tumour, accounting for approximately 50% of all benign cardiac tumours [1]. The clinical manifestations of cardiac tumours, such as heart failure, arrhythmias, valvular dysfunction and pulmonary embolism, are diverse and often lack specific symptoms, making them easily confused with other cardiac diseases. The location, size, shape and activity of cardiac tumours, as well as their relationship with surrounding tissues, significantly impact the haemodynamics of the heart. Therefore, early and accurate diagnosis, as well as effective treatment, are crucial for the prognosis of patients with cardiac tumours [2, 3]. Currently, surgical resection has been the preferred treatment for cardiac tumours [4], with a favourable prognosis and low recurrence rates having been observed for benign cardiac tumours. Most malignant cardiac tumours have a poor prognosis, are prone to recurrence and are often surgically treated for palliative purposes, providing temporary relief of symptoms. A large-scale study conducted by Hoffmeier *et al*. [5], which included 181 patients undergoing cardiac tumour surgery, revealed 5-year survival rates of 83%, 30% and 26% for benign, malignant and metastatic tumours, respectively.

With the continuous development and widespread use of cardiac imaging, incidence rates of cardiac tumours have significantly increased [6]. Multimodal imaging plays a crucial role in evaluating suspected cardiac tumours and aims to confirm their presence; describe their size, location and extent; and exclude the possibility of malignancy in order to provide optimal medical management. Key imaging techniques include transthoracic echocardiography, computed tomography (CT) and cardiac magnetic resonance imaging (CMR). In cases of suspected coronary artery obstruction, additional coronary angiography or coronary artery-enhanced CT should be performed to guide coronary artery management during surgery. Positron emission tomography (PET) can help differentiate between benign and malignant tumours, with a sensitivity exceeding 90% [7]. Three-dimensional (3D) printing technology has continued to advance in recent years such that image-based 3D-printed models have been incorporated into the auxiliary diagnosis and treatment of cardiac tumour diseases. Compared to traditional diagnostic imaging techniques, 3D printing technology allows doctors to print customized models based on the patient’s specific tumour anatomy, enabling doctors to visually observe and understand the location, size and shape of heart tumours so as to better plan surgical protocols and select the best treatment strategies. Doctors can simulate surgeries on 3D-printed models and familiarise themselves with surgical difficulties and risks in advance, thereby increasing the success rate and safety of surgery. 3D-printed models can be used as a teaching tool to help teams of doctors communicate better with each other, as well as facilitate in clearly explaining diagnosis and treatment plans to patients.

However, the application of 3D-printed models in the perioperative management of cardiac tumours remains controversial. This article aims to explore the current applications, advantages, challenges and future perspectives of 3D printing technology in the management of cardiac tumours during the perioperative period through a comprehensive review of relevant studies and analysis of clinical experiences.

## 2. Materials and Methods

This study followed the methodological framework presented in the PRISMA guidelines and conducted a literature search in the PubMed, Embase, Scopus and Google Scholar databases. The search was conducted up until March 2023. The search terms used for both the subject heading and keyword searches included ‘3D printing’, ‘three-dimensional printing’, ‘3D printer’, ‘rapid prototyping model’, ‘cardiac tumour’, ‘heart tumour’, ‘3D Surgical Planning’ and ‘cardiac surgery’. Additionally, the reference lists of relevant articles were searched. Two evaluators independently reviewed all the retrieved literature and screened them based on inclusion and exclusion criteria. In cases of disagreement, decisions were made through discussions or with the involvement of a third evaluator. The inclusion criteria consisted of studies involving the use of 3D printing technology in cardiac tumours, whereas the exclusion criteria comprised conference abstracts, editorials or review articles. Data collected included the first author, publication year, patient age and gender, 3D printer model, materials used, printing time and image sources. The surgical approach and follow-up information for patients whose treatment involved 3D printing technology were also recorded. Statistical analysis involved descriptive statistics for demographic and continuous data (e.g. mean ± standard deviation). Two-class variables were presented as numbers and percentages.

## 3. Results

**Fig. 1.**
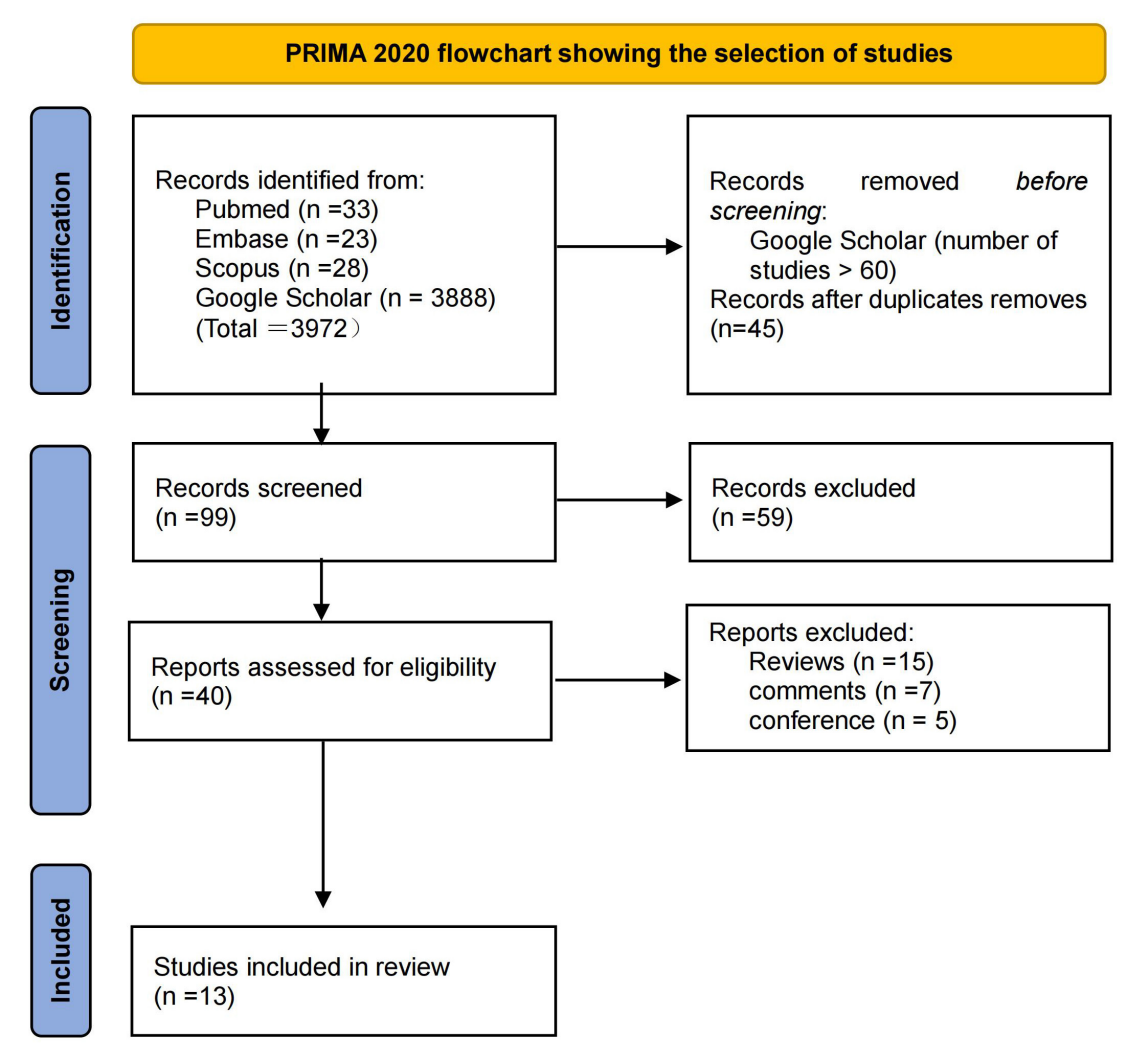
**PRISMA guidelines show study election process**.

After searching for relevant literature, a total of 33, 23, 28 and 3888 studies were obtained from the PubMed, Embase, Scopus and Google Scholar databases. Considering the large number of studies returned by Google Scholar, the search contents were sorted according to relevance, and we only paid attention to the results of the first 6 pages (10 studies each) of studies, which were highly relevant to our topic, while most of the following contents were irrelevant to the topic. Therefore, we selected 60 studies from these six pages for analysis. In the four databases searched, a total of 45 articles were duplicates and duplicates were deleted. Among the 99 articles identified, thirteen specifically focused on the application of 3D printing in cardiac tumours and were included in the scope of analysis. The publication dates of these articles ranged from 2008 to 2022. In cases where the same author had multiple relevant papers, only the most recent one was analysed. Fig. 1 illustrates the results of the literature search conducted according to the PRISMA guidelines.

### 3.1 Characteristics of Included Literature

**Fig. 2.**
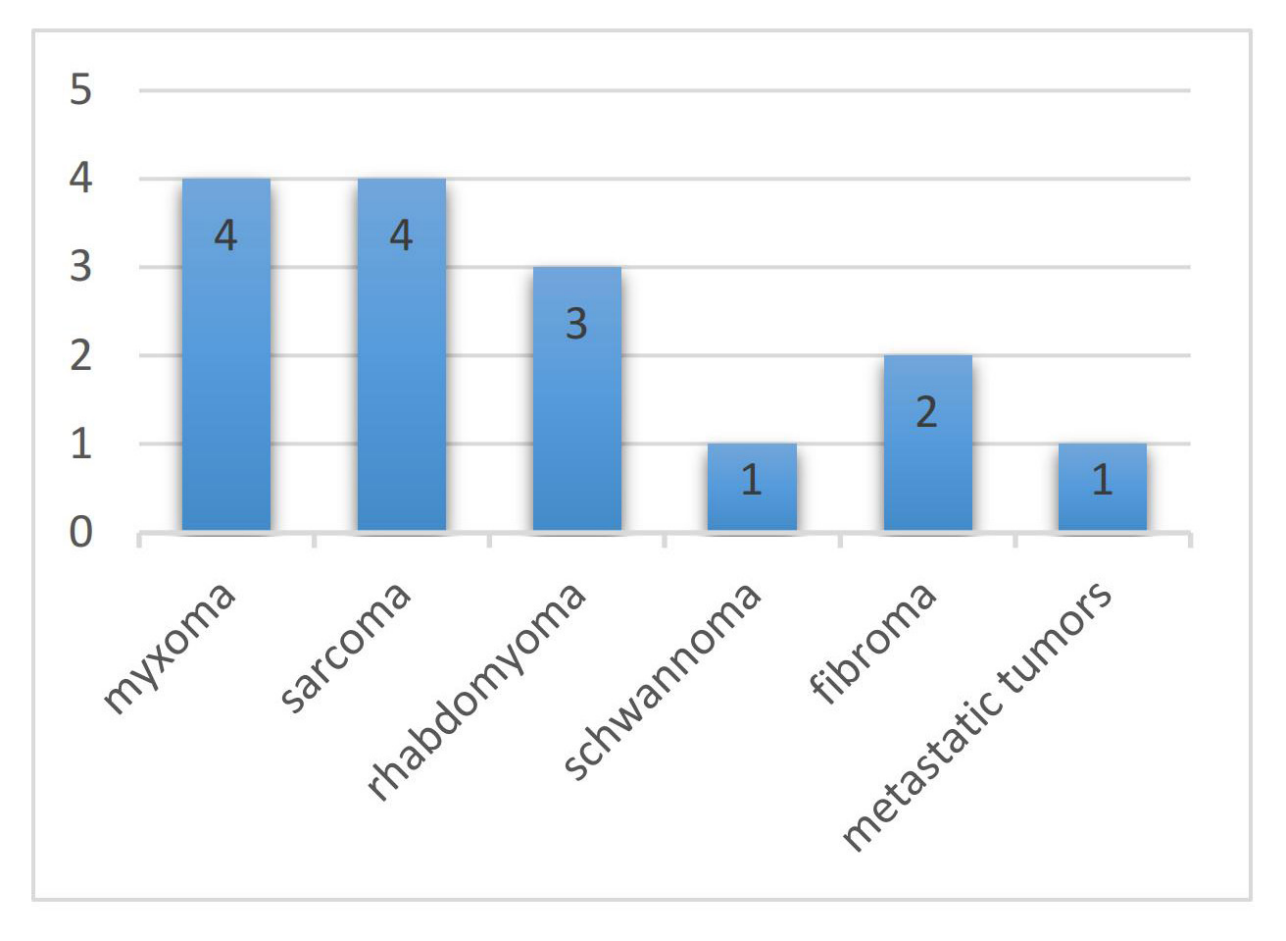
**Tumour types studied for preoperative planning of cardiac tumours using three-dimensional (3D)-printed models**.

In these 13 studies, 3D printing applications in cardiac tumours were presented in the form of case reports. We reviewed a total of 16 patients given that two cardiac tumour cases were reported in three studies. Table 1 (Ref. [8, 9, 10, 11, 12, 13, 14, 15, 16, 17, 18, 19, 20]) summarizes the characteristics of the eligible studies in this review. Fig. 2 illustrates the tumour types studied for preoperative planning of cardiac tumours using 3D-printed models, with one report not mentioning the pathological category of the tumour [11]. Mucinous and sarcomatous tumours were the most frequently reported, followed by rhabdomyomas. The average age of the patients was 34.4 ± 24.1 years, with durations less than 1 month calculated as one month. One study (two cases) did not mention the age of the patients [12]. Among the studied patients, six were male, six were female and two were undetermined (one report did not provide the gender of the patients) [13]. Given that patients included in the case reports exhibited complex anatomical structures, difficulties were faced in distinguishing the relationship between the anatomy and surrounding structures with two-dimensional (2D) imaging. All reports concluded that patient-specific 3D-printed models can visually present complex anatomical structures before surgery, aid in preoperative surgical decision-making and provide intraoperative surgical guidance.

**Table 1.** **Characteristics of the eligible studies in this review**.

Authors	Year	Age (years) and gender	3D Printer	Materials	Tools	Printing time	Imaging technique	Tumour location, type and size (CM)	Surgical method	Follow-up time
Jacobs *et al*. [8]	2008	50/Female	3D printer $Z^{\text{TM}}$ 510	Plaster	Mimics 9.0 software (Materialise, Leuven, Belgium)	(Segmentation time) 0.5-mm CT data semi-automatically 3 h	CT	Right ventricular angiosarcoma	A radical tumour resection, a mechanical tricuspid valve replacement and an epicardial pacemaker	NR
						0.5-mm CT data manually 8 h. 1.0-mm CT data manually 5 h		NR		
Schmauss *et al*. [9]	2013	43/Female	NR	NR	NR	NR	CT	Right ventricular fibroma	Radical tumour resection	NR
								NR		
Son *et al*. [10]	2015	42/Female	uPrint (Stratasys Ltd., MN, USA)	NR	Mimics Base version 16 (Materialize, Leuven, Belgium)	NR	CT	Right atrium Schwannoma	Radical tumour resection	NR
								14 × 10 × 7		
Al Jabbari *et al*. [11]	2016	50/Male	Objet500 Connex3 printer (Stratasys Ltd., Eden Prairie, MN, USA)	NR	Mimics® Innovation Suite (Materialise NV, Leuven, Belgium)	NR	CT	Left atrium osteosarcoma	Cardiac autotransplantation, radical tumour resection	6 months
								5.4 × 3.5		
		67/Female						NR	Radical tumour resection	6 months
								3.0 × 3.0		
Golab *et al*. [12]	2016	56/Male	CB-Printer	NR	Index Copernicus International	22 h	CT	Right atrial renal cell carcinoma metastatic tumour	Radical tumour resection	NR
								NR		
Farooqi *et al*. [13]	2017	NR	NR	NR	NR	NR	MRI	Right ventricular fibroma	Radical tumour resection	NR
								6 × 4 × 3		
		NR					CT	Left ventricular rhabdomyoma	Conservative treatment, 3D model as a guide during catheter ablation	
								NR		
Riggs *et al*. [14]	2018	2 months/Female	Objet260 Connex3 (Stratasys, Ltd., Eden Prairie, MN, USA)	Opaque PolyJet resin (actual heart), rubber-like transparent material tumour(tumour)	Mimics Innovation Suite 3D visualisation software interface (Materialise, Inc., Leuven, Belgium)	NR	MRI	Right ventricular myxoma	Radical tumour resection	2 years
								NR		
		12 days/Male					CT	Left ventricular rhabdomyoma	Conservative resection of the tumour	NR
								NR		
Menegazzo *et al*. [15]	2019	20/Male	NR	NR	NR	NR	CT	Left atrial monophasic synovial sarcoma	Cardiac autotransplantation, radical tumour resection	1 year
								7.4 × 7.1		
Liu *et al*. [16]	2019	2 months/Male	NR	NR	NR	NR	CT	Left ventricular rhabdomyoma	Cardiac autotransplantation, radical tumour resection	5 months
								2.7 × 1.7 × 2.5		
Ali *et al*. [17]	2020	63/Female	Form 2 (Formlabs, Somerville, MA)	NR	Materialise Mimics inPrint 2.0 (Materialise NV, Leuven, Belgium)	NR	CT	Left atrial myxoma	Radical tumour resection	1 year
								3.0 × 3.0 × 2.5		
Zhou *et al*. [18]	2021	50/Female	Sailner J501 Series Color Multimaterial 3D printer	Photosensitive resin material	NR	5 hours	CT	Left atrial myxoma	Cardiac autotransplantation, radical tumour resection	9 months
								8.8 × 7.6		
Kim *et al*. [19]	2021	33/Female	Projet 460 printer	VisiJet PXL Core powder, VisiJet PXL clear binder and colour bonds	3D Systems, Rock Hill, SC	NR	CT	Right ventricular liposarcoma	Cardiac autotransplantation, radical tumour resection	NR
								6.6 × 7.0 × 4.0		
Qiu *et al*. [20]	2022	7/Male	J501 Pro (Sailner, Zhuhai, ChiNR)	NR	NR	NR	CT	Left ventricular myxoma	Radical tumour resection	15 months
								2.0 × 4.0		
								6.6 × 7.0 × 4.0		

NR, non-reported; CT, computed tomography; MRI, magnetic resonance imaging; ^TM^, trademark; ®, registered; CM, centimeter.

### 3.2 Clinical Applications of 3D Printing in Cardiac Tumours

The current review included 13 studies with imaging data sourced from both computed tomography (CT) or magnetic resonance imaging (MRI), but predominantly CT. The literature included in this study utilized 3D printing technology to assist in the resection of cardiac tumours. Among them, three studies utilized patient-specific 3D cardiac tumour models for preoperative planning and simulation, leading to alterations in the initial surgical approach and the selection of a more convenient and less damaging method for tumour removal [8, 10, 20]. In these studies, five patients underwent autologous cardiac transplantation for the resection of cardiac tumours, which can be considered for left-sided malignant tumours or complex benign cardiac tumours [11, 15, 16, 18, 19]. Among the 16 patients analysed, 8 had postoperative follow-up data. The average follow-up duration was 11.13 ± 5.88 months, during which no tumour recurrence was observed.

### 3.3 Materials and Techniques in 3D Printing

The present review encompasses a series of studies, four of which reported on the materials used in 3D printing, primarily Vero and Tango materials that successfully achieved distinct staining of heart models to differentiate between various anatomical structures. In terms of image data acquisition and processing, eight studies mentioned the use of Mimics software, which has been widely applied in this field. Additionally, three studies provided detailed insights into the time required for model fabrication. The production of a complex and large metastatic cardiac tumour took 22 h, whereas the manufacturing of a complex mucinous tumour required only required 5 h. Furthermore, Jacobs *et al*.’s [8] study indicated that printing time depended on the DICOM data source used (e.g., CT, MRI, or echocardiography) and the segmentation model technique. Semi-automatic segmentation with a 0.5 mm slice thickness CT dataset averaged around 3 h, whereas manual segmentation with a 0.5- and 1.0-mm slice thickness took an average of 8 and 5 h, respectively. Only the manual segmentation method with a 0.5 mm slice thickness demonstrated acceptable results in terms of target area and structure identification. Regarding economic costs, only one study described the expenses associated with printing a cardiac tumour model, with the cost of producing a metastatic right atrial tumour from renal cell carcinoma amounting to 100 euros [12].

### 3.4 Workflow for the 3D Printing of Cardiac Tumours

**Fig. 3.**
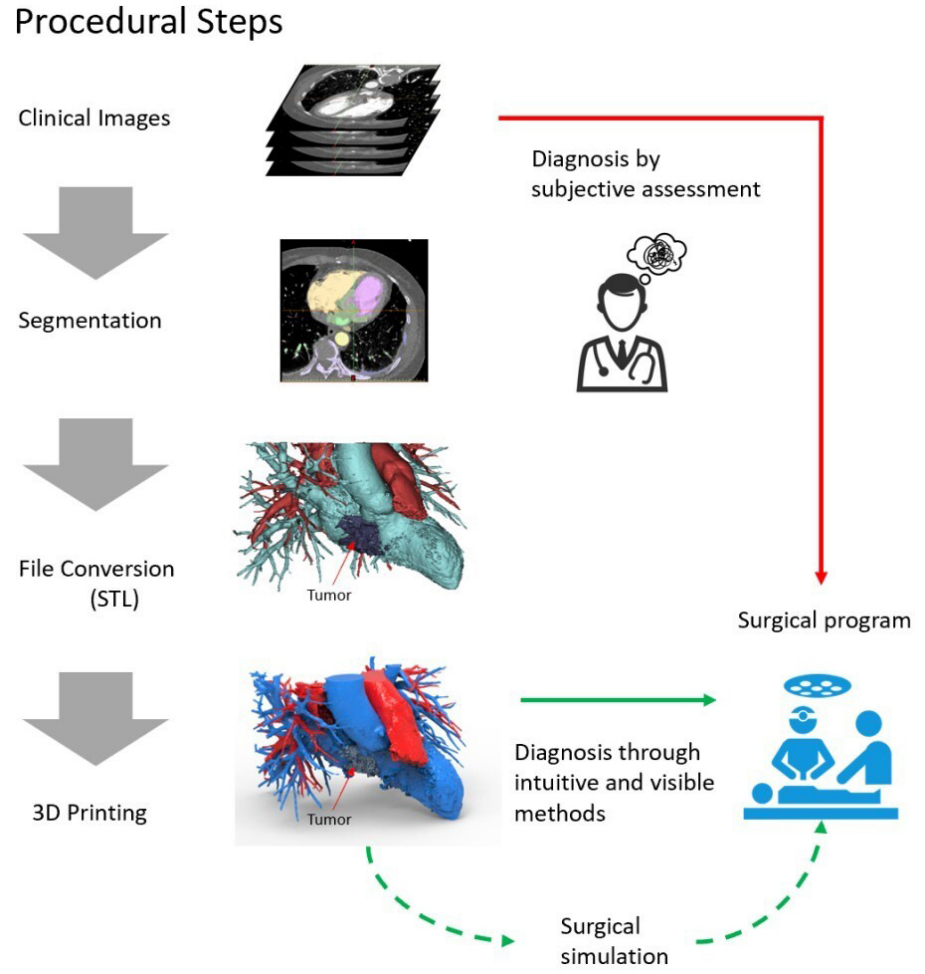
**Workflow for the 3D printing of cardiac tumours**. STL, Stereolithography; 3D, three-dimensional.

The 3D printing of cardiac tumours is a process that involves creating a physical model of the shape and structure of a cardiac tumour using 3D printing technology. The general workflow for the 3D printing of cardiac tumours is outlined below (Fig. 3).

#### 3.4.1 Collection of Medical Imaging Data

Medical imaging modalities, such as CT, MRI or ultrasonography, are used to scan the cardiac tumour and obtain detailed information regarding its structure and morphology. These imaging data will be used for subsequent model creation.

#### 3.4.2 Reconstruction and Segmentation

Medical imaging data are then reconstructed and segmented using image processing software. The purpose of this step is to separate the tumour from the surrounding tissues and generate a 3D model file suitable for printing.

#### 3.4.3 Model Design and Refinement

Based on the segmented 3D model, computer-aided design software is used for model design and refinement. Physicians or engineers can adjust the size, shape and details of the model as needed to best represent the characteristics of the cardiac tumour.

#### 3.4.4 File Preparation

The designed and refined model is converted into a file format suitable for 3D printing, such as Standard Tessellation Language. These files contain the geometric shape and surface information of the model, which are necessary for the subsequent printing process.

#### 3.4.5 Material Selection and Printing Parameters

Suitable 3D printing materials and printers are selected based on requirements and budget. The print parameters, such as layer thickness, fill density and print speed, are also determined at this stage.

#### 3.4.6 3D Printing

The prepared model file is printed using the selected 3D printer. During the printing process, the 3D printer stacks materials layer by layer according to the predetermined parameters, gradually forming a complete model of the cardiac tumour.

#### 3.4.7 Post-processing

After printing is completed, the necessary post-processing work is performed on the model, which may include removing support structures, performing surface smoothing, and applying colour painting or coatings to enhance the quality and appearance of the model.

Through the above process, 3D-printed models of cardiac tumours can be created for use in medical education, preoperative planning, patient education, research, and other areas, providing physicians and patients with a more intuitive and tangible means of understanding and communication.

## 4. Discussion

In the field of cardiovascular surgery, the application 3D printing technology throughout the perioperative period has seen rapid growth over the past decade. As an innovative technology in the medical field, 3D printing provides unique advantages for visualizing the complex anatomical morphology of patients with cardiac diseases, demonstrating enormous potential for assisting diagnosis during the perioperative period. 3D printing technology provides a whole new dimension to cardiac tumor surgery, improving the success rate of radical tumor removal through an individualized approach and precise navigation, and is expected to have a positive impact on long-term patient survival. This review demonstrates the accuracy and intuitiveness of 3D-printed models and emphasises the importance of their multiple roles in the perioperative management of cardiac tumours. These roles include assessing surgical risks and developing preoperative plans, conducting simulated surgeries, facilitating clinical teaching and improving doctor–patient communication.

### 4.1 3D Model-assisted Treatment of Cardiac Tumours

Cardiac sarcomas are a type of malignant tumour that have been extensively studied using 3D cardiac models, as evident from the literature reviewed herein. They are the most common malignant tumours affecting the heart, accounting for approximately 75% of all cardiac malignancies. These tumours are highly aggressive and rapidly invade the layers of the heart, causing widespread distant metastasis. Currently, surgical tumour resection has been considered the preferred treatment method [21]. In fact, research conducted by Kim *et al*. [22] demonstrated that cardiac sarcoma patients who did not undergo surgical treatment had a survival period as short as 1 month. In the current review, we report studies on four different types of cardiac sarcomas, namely vascular sarcoma [8], osteosarcoma [11], synovial sarcoma [15] and liposarcoma [19]. Among them, Jacobs *et al*. [8] utilised 3D-printed models for preoperative diagnosis, which facilitated the revision of the surgical plan such that the surgical approach was changed from the right atrium to the right ventricle. Given the complex tumour structures and their unclear relationships with the surrounding anatomical structures in the remaining three patients, autologous heart transplantation surgery was performed under the guidance of 3D-printed models to facilitate tumour resection. One patient with osteosarcoma was followed up for six months, during which no signs of tumour recurrence was detected, whereas the other three patients had no data on the follow-up duration. Therefore, we believe that preoperative 3D-printed cardiac tumour models customised based on patients’ imaging data are worth considering to successfully achieve radical resection of cardiac sarcomas with complex anatomical structures. This approach allows for a more intuitive evaluation of the relationship between cardiac tumours and other anatomical structures, thereby facilitating autologous heart transplantation surgery and addressing the challenge of achieving a clear resection of in-situ tumours.

The present review found that cardiac myxomas were the most common type of cardiac tumour, accounting for 75% of all cardiac tumours [23]. Although myxomas can occur at any location in the heart, they most commonly occur in the left atrium, which account for 72% to 92% of cases [24]. The current review included four cases of myxomas, of which two were located in the left atrium, one in the right ventricle and one in the left ventricle. In a case reported by Ali *et al*. [17], 2D imaging revealed that the myxoma potentially involved the superior vena cava, aorta and right upper pulmonary vein. In this particular study, a separate printing technique was employed to print the individual components of the model (tumour, aorta, left atrium, right atrium and right pulmonary artery). Acrylic paint was used for staining the 3D models to differentiate between different structures, while magnets were utilised to assemble the components together for the accurate identification of anatomical structures. In a case of right ventricular myxoma reported by Riggs *et al*. [14], preoperative MRI revealed an unclear border between the tumour and the coronary artery, leading to the creation of a 3D-printed model. The 3D model demonstrated that the tumour could be separated from the right coronary artery but surrounded the left anterior descending coronary artery. Guided by the model, however, the researchers were able to successfully and safely removed the tumour, which was not visible on the MRI images. With the aid of the 3D model, surgeons can better understand the anatomical relationships and assess the feasibility of safely removing the tumour without compromising the coronary arteries. In the treatment of a patient with left ventricular myxoma, Qiu *et al*. [20] combined the 3D-printed model with virtual reality to further enhance the realism of the preoperative surgical simulation, which prompted a change in the surgical approach from the mitral valve to the aorta for a more convenient tumour resection. Overall, despite being the most common type of benign tumourcardiac tumour, myxomas are complicated to remove due to factors such as abnormal tumour location, unclear anatomical structures and relatively young patients. In such cases, the use of 3D-printed models can assist in diagnosis and surgical planning.

The current review incorporates three studies on cardiac rhabdomyomas located in the left ventricle. Cardiac rhabdomyomas, which are hamartomas composed of myocardial cells, represent the most common pathological type of primary cardiac tumour in children, accounting for approximately 50%–60% of primary cardiac tumour cases in such populations [25]. All three cases included in the current review involved infants who had just been born, posing certain challenges for surgery due to their small hearts. In one case reported by Farooqi *et al*. [13], a child with a large cardiac rhabdomyoma experienced partial left ventricular outflow obstruction and malignant ventricular arrhythmias. The study employed a 3D-printed model to guide catheter-based radiofrequency ablation for the treatment of ventricular arrhythmias and utilised the 3D model for surgical planning. The researchers noted that the tumour occupied a significant portion of the left ventricular and that its excision could potentially cause severe cardiac dysfunction, prompting them to consider heart transplantation. In the second case reported by Riggs *et al*. [14], the tumour was massive and exerted pressure on the bronchus. Moreover, the relationship between the tumour and the coronary arteries was unclear, which increased the surgical complexity. Through meticulous planning using a 3D model, the researchers managed to remove a significant portion of the tumour without damaging the coronary arteries, thereby alleviating the airway obstruction. However, the patient in this case succumbed to multiple organ failure caused by postoperative infection. Although the child did not survive, the researchers discussed that this outcome might have been attributed to the severity of the case and the young age of the patient rather than the limitations of the 3D-printed model. They still supported the use of 3D-printed models in the preoperative planning for complex cardiac tumours considering its significant assistance in comprehending intricate cardiac structures. In summary, rhabdomyomas can regress spontaneously in some children. For larger tumours causing obstruction and with complex locations, surgical resection can be performed under the guidance of 3D-printed models.

Two cases of cardiac fibroma had been reported in the studies analysed herein. In one case, Farooqi *et al*. [13] utilised patient MRI data, which was a less commonly used data source in the current review, to create a cardiac model. MRI has advantages in imaging soft tissues, providing images with higher contrast and anatomical details and displaying soft tissue structures of the heart such as the myocardium, cardiac chamber wall thickness and pericardium. This makes MRI considerably useful for observing the internal structures of the heart and cardiac pathological processes. In contrast, CT is more effective in visualising calcifications and highlighting hard tissue structures, such as calcified plaques [26]. Currently, no research has compared the differences between MRI and CT and established their respective indications in creating 3D models of cardiac tumours. The specific choice of technique still depends on the physician’s assessment of the patient’s specific condition and clinical needs. Primary cardiac schwannomas are relatively rare, with only one related study having been included herein. In this particular study, Son *et al*. [10] reported that it was difficult to confirm the origin of the schwannoma from the heart or mediastinum based on 2D images obtained preoperatively. Guided by a 3D-printed model, the researchers believed that a sternotomy approach and tumour resection under cardiopulmonary bypass were more appropriate. Therefore, the researchers concluded that 3D printing was helpful in determining the surgical approach.

In summary, 3D printing, by providing precise preoperative planning and individualized surgical strategies, coupled with intraoperative navigation and real-time adjustments, assists surgeons in accurately locating and completely excising tumors during cardiac surgeries. In cases requiring the removal of substantial cardiac tissue, 3D printing technology can be utilized to manufacture personalized implants for reconstructing cardiac structures, thereby reducing the occurrence of postoperative complications. Consequently, 3D printing technology holds the potential to positively impact long-term survival rates for patients.

### 4.2 Promoting Physician–patient Communication and Clinical Education

Several of the studies included in this article have reported that 3D printing technology played a significant role in promoting physician–patient communication and clinical education [11, 14, 15]. First, 3D printing technology promotes physician-patient communication by allowing doctors to effectively communicate complex medical information to patients through the visualisation of cardiac tumours. By transforming medical imaging data into 3D-printed models, doctors can visually present the tumour’s location, infiltration patterns and relationship with surrounding structures in a tangible and easily comprehensible form. This visual aid enhances patients’ understanding, promotes shared decision-making and strengthens the doctor–patient relationship. Patients can actively participate in discussions, raise questions and make informed choices regarding suitable treatment options based on their understanding of their own condition [27]. Second, 3D printed models advance clinical education by enabling the accurate depiction of complex cardiac anatomy and pathology associated with tumours, providing a convenient means for a more immersive learning experience. Medical students and practicing physicians can study and manipulate 3D-printed models to gain a deeper understanding of tumour characteristics, anatomical variations and surgical techniques [28].

### 4.3 Comparison between Techniques for 3D Printing Cardiac Tumour Models

In the current review, the most commonly used printer for 3D printing heart tumour models was the stereolithography (SLA) 3D printer [17, 18, 19]. SLA 3D printing technology utilises photosensitive resins as materials and employs ultraviolet light or other light sources to selectively solidify the resin layer by layer, thereby creating intricate objects. SLA 3D printers are widely applied in printing heart tumour models given their high precision and ability to depict fine details. They can produce models with complex structures and subtle features, accurately replicating the shape and tissue structure of tumours. Additionally, SLA 3D printers have a fast printing speed, allowing for a relatively succinct printing process [29, 30]. Of course, with the continuous advancement of technology, other types of 3D printing techniques for printing heart tumour models have emerged. For example, PolyJet and ColorJet 3D printers can be employed for such applications and may have advantages over SLA printers in certain printing requirements [31].

Several factors can influence the printing time and cost of 3D-printed heart tumour models, including the size, complexity, printing material and 3D printing technology used for the models. First, the size of the model is one of the key factors affecting printing time and cost. Smaller models generally require less printing time and cost, whereas the opposite is true for larger models. For instance, Golab *et al*. [12] reported a printing time of 22 h for a model of a right atrial metastatic tumour from renal cell carcinoma, which is significantly longer than the 5h reported by Zhou *et al*. [18] for printing a left atrial myxoma. Therefore, the size of the heart tumour model directly impacts the required printing time and cost. Second, the complexity of the model is also an important factor. Evidently, models that require a higher resolution, more detail or a more complex structure would be associated with increased printing time and cost. Next, different 3D printing materials have different costs. For instance, using common plastic materials like polylactic acid or polymers is cheaper than using resinous materials. The choice of material would certainly affect the total cost based on the desired model quality and requirements. Finally, the 3D printing technology used also affects the printing time and cost. Different 3D printing technologies have different printing speeds and costs. For example, SLA 3D printing is typically faster than fused deposition modelling (FDM); however, this also depends on the specific equipment and printing parameters used. Using traditional FDM technology may be cheaper than using selective laser sintering or SLA technologies. Typically, the printing time for small heart tumour models may range from a few hours to a dozen hours. For larger and more complex heart tumour models, the printing time may take several tens of hours or even longer. The cost of 3D-printed heart tumour models ranges from tens to hundreds of dollars, depending on the combined impact of the aforementioned factors. However, it is important to note that only the printing process time has been discussed here, excluding the model design and preparation stages. These steps may require additional time, depending on the complexity of the model and software tools used.

Currently, several different software programs can be used for 3D printing heart tumour models. However, the current review will focus on the most commonly used printing software, Mimics [8, 10, 11, 14, 17]. Mimics is a medical image processing software that has been extensively employed in the field of medicine for 3D reconstruction and visualisation. When creating 3D-printed heart tumour models, Mimics can assist doctors or researchers in extracting the structures of interest, such as heart tumours, from medical imaging data and transforming them into 3D models. Moreover, this software enables a series of tasks including image preprocessing, region segmentation, 3D reconstruction, model editing and model exportation. However, despite being a powerful software, Mimics may require a certain amount of learning and practice prior to usage. For complex heart tumour models, the assistance of medical imaging experts or experienced technical personnel may be necessary for processing and operation [32].

### 4.4 Limitations in the Application of 3D Printing for Cardiac Tumours

The 3D printing of cardiac tumour models is an emerging and promising technology that helps physicians and researchers better understand and study cardiac tumours. However, several challenges in the development and utilisation of this technology do still exist, which we highlight in the following discussion. First, the creation of 3D-printed cardiac tumour models still requires a significant amount of non-automated manual work. For instance, Riggs *et al*. [14] emphasised the complexity of tumour image segmentation and 3D reconstruction in 3D printing studies, requiring specialised technical training. This process necessitates close collaboration among radiologists, surgeons and modelling engineers to avoid model distortions. The complexity of manual image processing procedures also contributes to a longer overall time for 3D printing models, making it less applicable in emergency cardiac tumour surgeries. Currently, highly accurate fully automated image segmentation and reconstruction algorithms are continuously being improved and developed, which hold the potential to enhance model accuracy and reduce image processing time in the future [33]. Second, 3D-printed models may not capture relevant information regarding the stalk of cardiac tumours. Jabbari *et al*. [11] demonstrated that the use of CT or MRI images for 3D-printed models has made obtaining more accurate anatomical information, such as tumour stalks, challenging given that such information may not be clearly visible on CT or MRI images. Therefore, acquiring high-quality medical imaging data for creating accurate 3D-printed cardiac tumour models is also a challenge. Third, the cost associated with producing these models can impose significant limitations on their utilisation. Manufacturing high-quality 3D-printed cardiac tumour models often requires expensive equipment and materials. Therefore, hospitals currently utilising this technology for tumour surgery planning are primarily research-oriented hospitals or research centres [34]. In the current paper, only one reference reported the costs of 3D-printed models [11]. Hence, reducing printing costs remains a major challenge for its widespread clinical adoption as a routine diagnostic and therapeutic tool. Fourthly, there is a current lack of large-sample controlled trials to demonstrate whether 3D printing improved clinical safety and long-term outcomes in patients with cardiac tumours. In fact, Jacobs *et al*. [8] stated that although existing literature indicates that 3D-printed cardiac tumour models improved preoperative planning accuracy, objective data to determine their beneficial impact on patient prognosis has been lacking. Despite these challenges, the potential of 3D-printed cardiac tumour models remains substantial. As technology continues to advance and improve, these challenges will gradually be addressed, aiding physicians in better understanding and managing cardiac tumours in terms of diagnosis, surgical planning and medical education.

### 4.5 Future Perspectives

Cardiac tumours present unique challenges given their complex anatomical location and diverse characteristics. Traditional diagnostic imaging techniques, such as CT and MRI, have limited ability to visualise and understand tumour infiltration and complex structures. However, 3D printing technology offers a revolutionary approach by creating patient-specific cardiac models with enhanced accuracy and realism. As such, future developments in 3D printing technology have the ability to disrupt the field of cardiac tumour management in several aspects. First, imaging data currently used for 3D printing are derived from a single data source. In the future, combining multimodal imaging data, including advanced techniques such as ultra-high-resolution MRI and positron emission tomography-computed tomography (PET-CT), with 3D printing may allow clinicians to generate more accurate cardiac models that depict tumour localisation, infiltration patterns and relationships with adjacent structures [35]. Such detailed models will aid in precise preoperative planning and achieving optimal tumour resection while minimizing the risk of complications. Second, the emergence of 3D printing opens new avenues for personalised treatment strategies in cardiac tumour management. By utilising patient-specific 3D-printed models, clinicians can simulate surgical interventions and explore various treatment options, including virtual tumour resection and reconstructive surgery [36]. Qiu *et al*. [19] have already used a combination of virtual reality and 3D models to assist in preoperative planning and simulated surgery, as described in the current review. This personalised approach further enhances the precision of surgical interventions, thereby improving patient outcomes and reducing surgical risks. Additionally, patient-specific 3D-printed implants and devices have the potential to revolutionise cardiac tumour treatment by enabling fully tailored solutions adapted to individual anatomy [37]. Third, to fully harness the potential of 3D printing technology in the field of cardiac tumour management, interdisciplinary collaboration and data sharing are crucial. Surgeons, radiologists, engineers, material scientists and others should work together to establish standardised protocols for 3D printing workflows, quality control and material selection [38]. Collaborative efforts will facilitate the integration of 3D printing technology into routine clinical practice, enabling seamless communication and optimizing patient management. Fourthly, technical advancements are needed to improve printing speed, resolution and material biocompatibility [39]. Additionally, the establishment of sound regulatory guidelines and ethical frameworks is crucial to ensuring patient safety and privacy in the application of 3D printing [40]. Long-term clinical research and cost-effectiveness analyses are also necessary to establish the efficacy and value of 3D printing in cardiac tumour management [41]. In conclusion, 3D printing technology holds tremendous potential in the diagnosis and treatment of cardiac tumours. Ongoing research, innovation and collaboration are key to addressing challenges and driving the integration of 3D printing technology into routine clinical practice, thereby improving the prognosis of cardiac tumour patients.

## 5. Conclusions

In recent years, 3D printing technology has demonstrated significant potential for application in the field of cardiac tumours. This paper comprehensively reviews of the application of 3D printing technology in the diagnosis and treatment of cardiac tumours. This paper highlights the advancements, challenges and future directions of this technology while emphasising its impact on personalised medicine, surgical planning and interdisciplinary collaboration. However, several challenges, such as the need for technological improvements and the lack of standardised guidelines and cost-effectiveness analyses, need to be addressed before 3D printing technology can be widely utilised in the management of cardiac tumours. We believe that in the near future, 3D printing technology will become a routine clinical diagnostic and therapeutic tool for cardiac tumours.

## Data Availability

All data and materials were from published researches.

## Abbreviations

3D printing, Three-dimensional; CT, computed tomography; MRI, magnetic resonance imaging; PET-CT, positron emission tomography-computed tomography; SLA, stereo lithography appearance; FDM, fused deposition modelling.

## Supplementary Material

Supplementary material associated with this article can be found, in the online version, at https://doi.org/10.31083/j.rcm2503101.
